# Ethnopharmacological Evaluation of* Breu* Essential Oils from* Protium* Species Administered by Inhalation

**DOI:** 10.1155/2017/2924171

**Published:** 2017-11-13

**Authors:** Eduardo Rodrigues da Silva, Danilo Ribeiro de Oliveira, Patrícia Dias Fernandes, Humberto Ribeiro Bizzo, Suzana Guimarães Leitão

**Affiliations:** ^1^Faculdade de Farmácia, Centro de Ciências da Saúde, Universidade Federal do Rio de Janeiro, Bloco A, 2° Andar, Sala 10, Cidade Universitária, 21941-902 Rio de Janeiro, RJ, Brazil; ^2^Instituto de Ciências Biomédicas, Centro de Ciências da Saúde, Universidade Federal do Rio de Janeiro, Bloco J, 1° Andar, Sala 10, Cidade Universitária, 21941-902 Rio de Janeiro, RJ, Brazil; ^3^Embrapa Agroindústria de Alimentos, Avenida das Américas 29501, Guaratiba, 23020-470 Rio de Janeiro, RJ, Brazil

## Abstract

**Background:**

* Breu* is an aromatic oleoresin which has been used by Amazonian traditional communities as a remedy for headaches and migraines by burning and inhaling the smoke produced during its combustion. This study evaluated the antinociceptive and sedative activities of formulations containing* breu* essential oils administered by inhalation.

**Methods:**

Five different formulations (A–E) containing* breu* essential oils were evaluated for their sedative and antinociceptive activities in mice. They were delivered for 20 minutes using an inhalation chamber coupled with a nebulizer and the air inside was collected by static headspace and analyzed by GC-FID.

**Results:**

All nebulized formulations had similar chemical compositions and major compounds as the original essential oils. None of them resulted in significant increase in response time during the hot plate test. In the formalin test, Formulation E showed a significant inhibition of licking responses in the early (46.8%) and late (60.2%) phases. Formulation B was effective (36.9%) in the first phase and Formulation D (37.9%) in the second. None of the formulations presented sedative effects.

**Conclusion:**

* Breu* essential oils, when inhaled, may present antinociceptive and anti-inflammatory properties without sedation. Additionally, nebulization proved to be an efficient method for administration of formulations containing these essential oils.

## 1. Introduction

Species belonging to the* Protium* genus (Burseraceae) produce a characteristic fragrant oleoresin with economic, medicinal, and cultural values [1, 2]. In the Brazilian Amazon region, these oleoresins are known as* breu, breu-branco* (white-tar), and* breu-preto* (black-tar) [3–5].* Breu* oleoresins are a combination of two fractions—a volatile fraction that is mainly composed of mono- and sesquiterpenes and a solid nonvolatile fraction that is mainly composed of triterpenes—and have a complex chemical composition with varying concentrations of each component responsible for* breu* therapeutic activities [6–9]. Despite traditional communities' belief that there is a difference between white and black* breu*, we have demonstrated that it is difficult to establish this nomenclature based on chemical, botanical, or regional names [5]. This traditional classification is probably associated with the darkening of the oleoresin caused by volatilization of select components and/or oxidation of others [5]. Among the mono and sesquiterpenes that characterize the volatile* breu* fractions, some present antimicrobial, antioxidant [10], analgesic [11], anti-inflammatory, and antitumor [12] activities. Because of its high sensorial quality,* breu* essential oil is also used as a fragrance in the cosmetic and pharmaceutical industries [13].

In March 2012, our research group embarked on an expedition to the* quilombola* territories along the Erepecuru River (Oriximiná, Brazil) in search of different* breu* trees and oleoresins to collect, analyze, and understand their use by the* quilombolas*. This journey was called “The Malungo Expedition” [5].* Quilombola* communities (descendants of African slaves) from Oriximiná, in the State of Pará, Brazil, use* breu* in their popular medicine to treat headaches and migraines by burning and inhaling the smoke produced during its combustion [14, 15]. It can also be used as a nasal decongestant by inhalation for severe colds [16, 17] as well as a topical treatment for contusions and inflammation and can be administered as a patch [18, 19] for colds, coughs, and bronchitis [3]. Furthermore,* breu* is employed for mystical, spiritual [16, 19, 20], and superstitious purposes [21] as well as for caulking boats [14, 20].

Since The Malungo Expedition [5], our group has been interested in evaluating the traditional uses of* breu* resins as a remedy for headaches and migraines by inhalation of the smoke produced during its combustion, but a literature search for pharmacological animals models resulted in no validated studies. Several pharmacological models of pain involve inflammatory mediators. We selected the formalin-induced licking model to evaluate inflammatory pain. This model also involves direct activation of nociceptors (via C-fibers). According to the* quilombola* tradition, the* breu* oleoresin is burned to generate smoke, which is inhaled. In this procedure, some of the original* breu* compounds may undergo pyrolysis reactions and some are simply transferred to the vapor phase and therefore inhaled without a chemical change. In the inhalation model developed for this study, we only tested the volatile fractions of the oleoresins because they had been fully characterized in previous work from our group. The present study was designed to explore the form of administration performed by the* quilombolas* and to investigate whether the compounds in* breu* essential oils, when included in an inhalation formulation, could produce a pharmacological response in* in vivo* antinociceptive and anti-inflammatory models. In addition, the sedative activity of these formulations was also evaluated to exclude a possible relationship with the observed pharmacological effects.

## 2. Materials and Methods

### 2.1. Chemicals and Drugs

Ethanol 96% (v/v) and propylene glycol were purchased from Spectrum (Spectrum Brasil, São Paulo, SP, Brazil).* Breu* essential oils* (Protium *spp*.)* were obtained by hydrodistillation from collected oleoresins and chemically characterized by HRGC-MS as described previously [5]. Distilled water was prepared in-house with a distiller.

### 2.2. Test Animals

Male Swiss* Webster* mice (20–25 g), donated by the Instituto Vital Brazil (Niteroi, Rio de Janeiro, Brazil), were used in this study. Animals were maintained under standard housing conditions (room with a light-dark cycle of 12 h, 22 ± 2°C, 60% to 80% humidity, and food/water provided ad libitum). Animals were acclimatized to laboratory conditions for at least 1 h before the onset of each test and were used only once throughout the experiments. Research was conducted in accordance with the internationally accepted principles for laboratory animal use and care as found in the European Community guidelines (EEC Directive of 1986; 86/609/EEC) and the US guidelines (NIH publication #85-23, revised in 1985). All protocols followed the principles and guidelines adopted by the National Council for the Control of Animal Experimentation (CONCEA), were approved by the Biomedical Sciences Institute/UFRJ, Ethical Committee for Animal Research, and received the number DFBCICB015–04/16. All experimental protocols were performed during the light phase. Animal numbers per group were kept at a minimum. At the end of each experiment, mice were euthanized by ketamine/xylazine overdose.

### 2.3. Preparation of the Inhalation Formulations

Glycoalcoholic solutions containing 0.1 g/mL* breu* essential oil, 10% (w/v) propylene glycol, and ethanol were prepared according to the popular use by the* Quilombola* Communities of Oriximiná (Pará, Brazil) [14]. According to* quilombola* knowledge, it is standard practice to burn and inhale approximately 20 g of* breu* oleoresin, with an average yield of 2.5% (w/w) of essential oil, which represents approximately 0.5 g of essential oil/20 g of oleoresin [5]. Therefore, a dose of 0.5 g of essential oil per 5 mL of the formulation was chosen (0.1 g/mL). The final formulation is described below, and it was prepared as follows: first, propylene glycol was homogenized with part of the ethanol, and, subsequently, essential oil was added under stirring. Finally, ethanol was added until the final volume (5 mL) was reached, and the solution was homogenized and stored in amber glass bottles under refrigeration (−4°C). Based on previous chemical characterization [5], essential oils from 10 different* breu* samples (Table 1) were mixed in equal parts for pharmacological tests, according to the similarity of their chemical compositions (major compounds) as follows: Formulation “**A**”, prepared with 0.125 g of BBIM, BBPIR, BBIR_1_, and BBIR_2_ essential oils, with *δ*-3-carene as the major compound; Formulation “**B**”, prepared with 0.25 g of BBIR_3_ and WBB1 essential oils, with* p*-cymene as the major compound; Formulation “**C**”, prepared with 0.25 g of BBTF1 and BBTF2 essential oils, with* p*-cymene as the major compound with high concentrations of sesquiterpenes; Formulation “**D**”, prepared with 0.5 g of WBB_2_ essential oil_,_ with limonene and *α*-terpineol as the major compounds; and Formulation “**E**”, prepared with 0.5 g of WBIG essential oil, with *α*-pinene as the major compound.

Final formulation was as follows:

 
*Breu* essential oil(s): 0.5 g
  Propylene glycol: 0.5 g
  Ethanol 96% (*q.s.p.*): 5.0 mL.

### 2.4. Essential Oil Administration by Inhalation in an Inhaling Chamber

**Figure 1:**
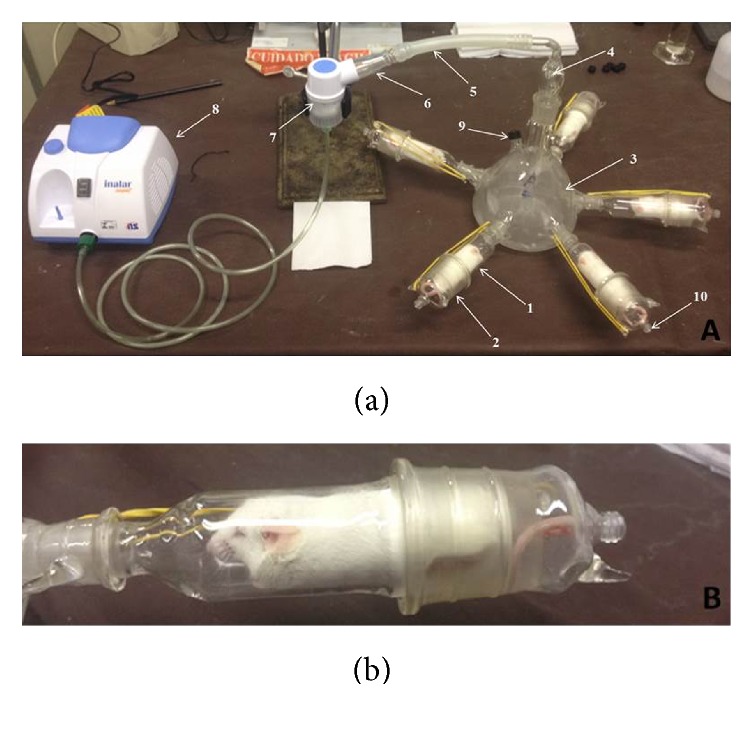
(a) Inhalation chamber coupled to a nebulizer. (1) Animal holder; (2) lid; (3) central part; (4) “head”; (5) silicone hose; (6) glass joint; (7) nebulizer cup; (8) nebulizer; (9) sampling port; (10) air output. (b) Animal holder with a mouse inside.

**Figure 2:**
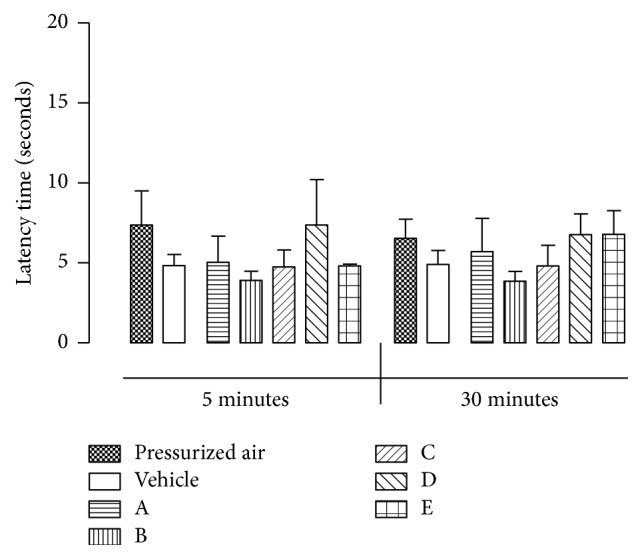
Effects of formulations on hot plate test in mice.* Each value is presented as the mean ± SEM *(*n* = 5).

**Figure 3:**
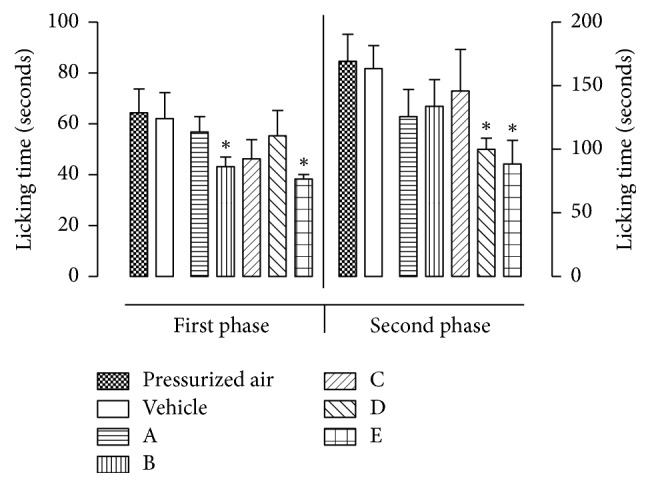
Effects of formulations on formalin-induced paw licking in mice.* Each value is presented as the mean ± SEM *(*n* = 5). *∗ indicates p < 0.05 compared with control groups (Bonferroni's test)*.

For each pharmacological test, seven groups containing five mice each were assembled. Each group was represented by mice that inhaled the following: nebulized air only (control group); the formulation vehicle without essential oil (vehicle group); and one of the formulations (A–E). The inhalation process was carried out in a chamber that was previously developed by our group (Figure 1) [22]. The chamber contains a central structure connected to five animal holders (Figure 1(a), 1) with lids (Figure 1(a), 2), and nebulized air passes directly and continuously through these animal holders. The central chamber (Figure 1(a), 3) has an outlet for air collection, known as a sampling port (Figure 1(a), 9), which has a threaded plastic cap with a septum to avoid air loss during* in vivo* tests. First, this chamber was assembled and coupled to the nebulizer cup by means of a silicone hose. Then, 5 mL of the test formulation, prepared as described above, was dispersed in a sufficient volume of purified water to a final volume of 15 mL and placed in the nebulizer cup. Animals were placed into the animal holders; the nebulizer was turned on and the formulation was nebulized into the inhalation chamber and each of the five animal holders simultaneously. The nebulized air was blown into the chamber from the head (Figure 1(a), 4) to the bottom by a central tube, generating a vortex that optimizes saturation and allows more uniform air distribution to the animals [22].

In all of the pharmacological tests, the inhalation lasted twenty minutes. All 15 mL were consumed.

### 2.5. Chemical Analysis of the Air Nebulized inside the Chamber

Chemical analysis of the nebulized air inside of the inhalation chamber was performed to assess the chemical composition of the volatiles inhaled by the animals. For analysis, the formulations were prepared as described above. The air inside of the chamber was collected (100 *μ*L) at 1 and 15 minutes after the nebulization started from the static headspace using a Hamilton Bonaduz AG syringe, Microliter™ Syringe, 2.500 *μ*L, and analyzed. Between each test, the chamber and nebulizer cup were washed with ethanol and water and dried.

Nebulized air relative compositions were obtained using gas chromatography coupled with a flame ionization detector (GC-FID) using two Agilent 7890 gas chromatographs. Separation was accomplished with a HP-5 fused silica capillary column (30 m × 0.32 mm i.d., 0.25 *μ*m phase thickness). The operating conditions were as follows: split ratio 1 : 10; injector temperature 250°C; carrier gas: hydrogen, 1.5 mL/min, constant flow; column temperature, 60°C (no hold), 3°C per min to 240°C; and detector temperature: 280°C. Between each analysis, the system was purged to avoid residual component carry over.

Linear retention indices were calculated by injection of a series of* n*-alkanes (C_7_–C_26_) [23] using the same column and conditions as described above for GC analyses. Peak identification was performed by comparison with chromatograms and retention times obtained previously by our group [5].

### 2.6. Hot Plate Test

Mice were tested to assess central antinociceptive activity according to the method described by Sahley and Berntson [24] and adapted by Matheus et al. [25]. Initially, all mice were evaluated for the determination of their individual baselines. Mice were placed over the stainless-steel heating plate that was at a temperature of 55 ± 0.1°C, and the time that it took for each mouse to remove one hind paw from the surface of the plate was timed. These verifications were taken 60 and 30 minutes before mice underwent inhalation. The baseline of each animal was calculated as the mean of the timed trials. The dwell limit time for each mouse was set at three times the baseline value. Baseline determinations were performed 2 days before the pharmacological test. After 2 days, mice were subjected to inhalation. Five and 30 minutes after the end of the inhalation test, mice were placed on the hot plate under same experimental conditions described above. The time that each mouse took to lift a hind paw was timed and scored for final analysis.

### 2.7. Formalin-Induced Licking Test

The peripheral analgesic and anti-inflammatory activities were evaluated using a model adopted by Hunskaar and Hole [26]. Immediately after inhalation, animals were withdrawn from the chamber and 20 *μ*L of a 2.5% (v/v) formalin solution in PBS was injected subcutaneously into the right hind paw. Animals were then transferred to a transparent acrylic box subdivided into six equal square areas. The time that mice licked their right hind paw in the first five minutes and from 15 to 30 minutes following completion of the inhalation test was timed.

### 2.8. Rota-Rod Test

Before evaluating the sedative activity of the formulations, mice were trained in the rota-rod apparatus twice for 10 minutes at 5 rpm with a 30-minute interval between training sessions. After the two training sessions, mice with an average number of falls equal to or greater than nine were eliminated from the test.

Two days after the training sessions, mice were placed in the chamber to inhale the formulations or controls. After inhalation, mice were removed from the chamber and placed in the rota-rod apparatus where they were tested for 5 minutes at 5 rpm immediately and 30 minutes after inhalation. In the training and testing sessions, the number of times that each mouse lost its balance and fell from the device during the period of time was counted [27].

### 2.9. Statistical Analysis

The* in vivo* experimental results are reported as the mean ± standard error of mean (SEM). Statistical analysis was performed using one-way analysis of variance (ANOVA) followed by Bonferroni's test for multiple comparison using SPSS 11.5 software. Differences between groups were considered significant at *p* < 0.05. 

## 3. Results and Discussion

### 3.1. Formulation Descriptions

Based on traditional* quilombola* medicinal use information, formulations containing* breu* essential oils were developed and evaluated for their antinociceptive and sedative activities. The final concentration of essential oil in the formulations (0.1 g/mL) was established based on the amount of* breu* used in burning and inhalation by* quilombolas* [14] as well as the mean yield of essential oil in the* breu* samples [5]. Although the traditional mode of use of* breu* by the* quilombola* communities involves burning and subsequent inhalation of the smoke produced, the chamber developed for this study does not foresee this procedure.

All of the formulations presented a clear and monophasic aspect, with a slightly yellowish color and a characteristic scent, indicating that solutions with molecular dispersion were obtained [28, 29]. Because they were extemporaneous formulations, it was necessary to disperse them in water before nebulization, generating a heterogeneous system known as a liquid-liquid dispersion. This probably occurs because the excess water makes the medium quite polar, preventing the apolar components of the essential oil from remaining in the solution [28, 29]. Despite the heterogeneous nature of the solution in the formulation cup, the compressed air injected by the nebulizer generates a vortex that guarantees homogeneous air nebulization inside the chamber [22].

### 3.2. Chemical Analysis of the Air Nebulized inside the Chamber

The chemical composition of the volatiles produced during operation of the nebulizing chamber was analyzed by static head space sampling of the chamber air by a sampling port. In this chamber, the animal holders have an air output (Figure 1(a), 10) that can be sealed by a plastic screw cap. These outputs are opened during the tests, forcing the intake air to leave the chamber thought them since they are the only exits from the chamber [22]. The main components in each essential oil formulation,** A**–**E,** were quantified in the nebulized air and are shown in Table 2. The chemical composition of each* breu* essential oil present in the formulations is described in Da Silva et al. [5]. Compared to the individual composition of the essential oils observed in a previous study [5], the composition of each nebulized formulation was quite similar to that of the original essential oils. In all formulations, the major components were present and at higher concentrations than the other components. A mixture of *δ*-3-carene/isosylvestrene was the major component of the head space air from Formulation “**A**” (58.96% in the first minute and 56.4% after 15 min), which was prepared with the BBIM, BBPIR, BBIR_1_, and BBIR_2_ essential oils (61.45% mean in the original oils);* p*-cymene was found at 20.6% (first minute) and 22.5% (after 15 min) in the head space air of Formulation “**B**”, prepared with the BBIR_3_ and WBB1 essential oils (32.7% mean in the original oils);* p*-cymene (27.9% in the first minute and 23.4% after 15 min) and a high concentration of sesquiterpenes were the major compounds in the nebulized air from Formulation “**C**”, prepared with the BBTF1 and BBTF2 essential oils (11.45% mean of *p*-cymene in the original essential oils); a mixture of limonene/*β*-phellandrene (33.4% in the first minute and 6.4% after 15 min) and *α*-terpineol (15.4% in the first minute and 68.9% after 15 min) was detected as the major component in the head space air of Formulation “**D**”, prepared with the WBB_2_ essential oil (41.1% limonene/*β*-phellandrene mix and 30.9%  *α*-terpineol in the original essential oil). Finally, *α*-pinene was found at 68.8% (first minute) and 49.2% (after 15 min) in the head space of Formulation “**E**”, prepared with the WBIG essential oil, which contained 57.7%  *α*-pinene in its original composition. From Table 2, it can be seen that, in the first minute, lower molecular weight components (monoterpene hydrocarbons) were detected at higher relative percent concentrations, as expected. This is why *α*-pinene was detected at a higher relative percentage in Formulation E in the first minute (68.8%) than in the original essential oil (57.7%). By the fifteenth minute, the relative percent concentrations of the monoterpene hydrocarbons decayed while concentrations of the oxygenated monoterpenes and sesquiterpenes increased. This suggests that initially mice receive a dose that is rich in monoterpenes, which are lighter and probably more easily dispersed in the nebulized droplets. As the formulation is depleted, it is heavier and more difficult to disperse components that are nebulized, increasing their concentration inside the chamber. This is clearly seen in Formulation** D**, in which the major compounds in the first minute are a mixture of *β*-phellandrene-limonene (monoterpene hydrocarbons), which are replaced by *α*-terpineol (oxygenated monoterpene) as the major volatile compound of the air sample collected at the second time point (15 min).

### 3.3. Hot Plate Test

The hot plate test was employed to assess antinociceptive activity. In the hot plate test, the response to pain stimuli is relayed to the supraspinal reflex mediated by *μ*_1_ and *μ*_2_ opioid receptors [30]. This test was developed by Woolfe & Macdonald in 1944 [31] and improved by many other researchers, such as Eddy et al. [32] and O'Callaghan and Holtzman [33]. In all of these cases, antinociceptive activity is characterized by an increased tolerance to pain by the animal when in contact with a heated plate.

None of the mice that inhaled any of the tested formulations showed a significant increase in response time compared to animals that inhaled compressed air or vehicle, indicating the absence of antinociceptive activity (Figure 2 and Table 3). This result is in accordance with previous work from Rao and coworkers [11], who reported that oral administration of* breu* essential oil obtained from* P. heptaphyllum* did not result in antinociceptive activity in the hot plate test.

### 3.4. Formalin-Induced Licking Test

Intraplantar administration of formalin produces nociception, which is characterized by two distinct phases [30]. The early phase (neurogenic phase) occurs in the first five minutes and is associated with direct chemical stimulus of the afferent fibers, mainly C-fibers [34, 35], with activation of TRPA1 channels [36], and reflects centrally mediated pain. The late phase (inflammatory phase) occurs between 15 and 30 minutes after formalin injection and is mediated by the release of a combination of inflammatory mediators and sensitization of central nociceptive neurons [26, 35, 37]. It is well-known that centrally acting drugs, such as opioids, inhibit nociception in both phases, while peripheral-acting drugs, such as acetylsalicylic acid, inhibit only the second phase [26, 38]. In addition, the activity of nonsteroidal anti-inflammatory drugs is also observed in the second phase [26, 34, 35].

Figure 3 and Table 4 show the results of the five formulations on the formalin test. The vehicle did not produce significant inhibition of the licking response in the early or the late phase. The results obtained from inhalation of the nebulized formulations were compared with those obtained from inhalation of the vehicle. Formulation B reduced the licking time significantly only in the early phase (36.9%), suggesting possible central pain inhibition. Formulation D suppressed the licking time significantly only in the late phase (37.9%), indicating possible peripheral antinociception by decreasing tonic inflammatory pain. Only Formulation E significantly suppressed the licking time in both the early phase (46.8%) and late phase (60.2%), indicating that it is effective on both tonic inflammatory and central pain.

Because the pain mechanisms assessed using the hot plate and formalin tests are different, it is common for substances to be active in only one of the models. This was observed with the oral administration of* breu* essential oil, which resulted in antinociceptive activity in both the capsaicin and formalin (only in the second phase) tests and none in the hot plate test [11]. This was also observed in the present study, as well as in others [39, 40]. The active substances present in all of these studies probably do not have affinity with opioid receptors, as they are inactive in the hot plate test.

The activity shown by formulations B, D, and E is related to their essential oil compositions since the compressed air (control group), vehicle (vehicle group), and Formulations A and C did not present a decrease in the licking time in either phase. Thus, the predominant presence of monoterpenes (*α*-pinene,* p*-cymene, *α*-phellandrene, limonene, and *β*-pinene) in the fraction of nebulized and inhaled air of Formulations B, D, and E (Table 2) may be related to the antinociceptive activity. In addition, the fraction of nebulized and inhaled air of Formulation E also had a high concentration of *α*-terpineol (Table 2), a potent analgesic that acts on both central and peripheral pain [41]. Interesting results regarding the antinociceptive activity of these monoterpenes administered by different pathways can be found in the literature. When administered intraperitoneally in mice, limonene presents antinociceptive activity in acetic acid and formalin (mainly in the second phase) tests without sedative properties [42]. Additionally,* p*-cymene injected intraperitoneally in mice presented orofacial antinociceptive activity in formalin, capsaicin, and glutamate tests, without sedative properties [43]. Similarly, *α*-phellandrene [39] and *β*-pinene [44] also showed antinociceptive properties in different models. These data suggest that the combination of these components in the formulations is a crucial factor in the observed antinociceptive activity.

In addition to antinociceptive properties, these monoterpenes present anti-inflammatory activity. Limonene, for example, in addition to being the major component of essential oils from* Citrus* species with anti-inflammatory activity when administered orally also had the same effect when tested alone [45]. Bergamot essential oil is rich in limonene and *α*-pinene, which are mainly responsible for the anti-inflammatory activity of Bergamot in the carrageenan test [46]. *α*-Pinene is also a major component in* Chenopodium album *L. [47] and* Ugni myricoides* [48] leaf essential oils, which have anti-inflammatory properties against TPA and carrageenan, respectively. This monoterpene is involved in immunologic activation and inflammatory intermediate synthesis inhibition, which are important for the pharmacological properties of many essential oils [49].

The different tested* breu* formulations significantly decreased the duration of the licking time in both phases of pain responses in the formalin-induced licking model. One possible explanation for this effect is that* breu* may act by decreasing the release of inflammatory mediators or exert direct effects on different receptors present in the paw, such as bradykinin, serotonin, or opioid receptors, thus reducing the licking response.

### 3.5. Rota-Rod Test

The rota-rod test is a safe and efficient test to assess an animal's motor coordination and balance. It has been employed to directly measure the influence of essential oils and their components on the central nervous system [50, 51]. Another advantage of the rota-rod test is that it allows distinguishing analgesic or anti-inflammatory effects from possible sedative effects since nonspecific muscle relaxation effects may reduce motor coordination and mask the mice's response to nociception [30].

Although* quilombolas* reported mild drowsiness and relaxation after burning and inhalation of the* breu* oleoresin [14], all of the tested animals remained conscious and no visible effects on their behavior were observed from* breu* formulation inhalation. The amount of alcohol in the formulation did not appear to affect the behavior of the animals since administration of the vehicle alone (which contains alcohol) did not generate any behavioral changes. Although it is well-known that several monoterpenes can induce seizures [52], we did not observe this effect in the tested mice.

None of the formulations with essential oils presented sedative or motor coordination depressant effects after nebulization and inhalation since the number of animal falls was quite reduced (Table 5). However, it should be taken into account that the* breu* formulations evaluated in the present study only contained the volatile fractions (essential oils) and that* breu* was not submitted to combustion. Burning of an oleoresin, as is usually done in the traditional use, may induce changes in the chemical composition, as pyrolysis may occur. This effect was not evaluated here. However, during the traditional mode of use, burning occurs slowly and at least some of the volatile compounds pass to the vapor phase by evaporation due to the heating flux, before pyrolysis, similar to cigarette burning, for example. Therefore, both the original compounds and pyrolysis products might be present in the smoke inhaled during traditional use. Nevertheless, the absence of sedative or motor coordination depressant effect is a satisfactory result since it demonstrates that any of the observed anti-inflammatory or antinociceptive effects are associated with a depressor effect above the central nervous system. Other essential oils, such as* Chrysopogon zizanioides* [39] and* Croton sanderianus* [40], also have antinociceptive and anti-inflammatory activity without sedative properties. Myrcene and linalool, which are components of the* breu* essential oil, have sedative and motor system depressant activities at high concentrations [53, 54]. However, it appears that their concentrations in the nebulized and inhaled air fractions were not enough to trigger such effects.

## 4. Conclusion

Experimental data on the pharmacological activity of* breu* essential oils administered by inhalation, in a similar manner as used in Amazon* quilombola* traditional communities, are presented for the first time. Nebulization of formulations containing* breu* essential oils provides a mixture of major* breu* components similar to those found in the pure essential oil and proved to be an efficient method of administration. Depending on the type and concentration of the components, the* breu* essential oils may present antinociceptive properties without sedation when inhaled after nebulization. The presence of monoterpenes, such as *α*-pinene,* p*-cymene, *α*-phellandrene, limonene, *β*-pinene, and *α*-terpineol, may play important roles in these bioactivities. Formulation E, which contains a high concentration of *α*-pinene, appeared to be the most promising formulation since it was the only formulation with significant activity in both phases of the formalin test. It is interesting to note that, with the exception of Formulation E (which suppressed licking time by 60.2% in the late phase), no activity was greater than 50% in the models tested. However, it is necessary to remember that* breu* essential oil formulations were administered by inhalation and not by common routes, such as oral or intraperitoneal routes, that present more pronounced effects. It is possible that, when administered by inhalation, the effects are not as prominent due to rapid elimination (via respiration). In this work, we demonstrated that formulations containing essential oils from* breu* samples can produce* in vivo* antinociceptive and anti-inflammatory activities when inhaled by nebulization.

## Figures and Tables

**Table 1:** Sample codes, identified species for each collected sample [5], formulation's composition, and major compounds in the essential oils of each formulation.

Sample code	Common name	Identified species	Formulation	Major compounds
BBIM	Black *breu* or *breuzinho*	*Protium heptaphyllum* (Aubl.) Marchand	A	*δ*-3-Carene
BBPIR	Black *breu*	*Protium decandrum *(Aubl.) Marchand		
BBIR_1_	Black *breu*	*Protium heptaphyllum* (Aubl.) Marchand		
BBIR_2_	Black *breu*	*Protium heptaphyllum* (Aubl.) Marchand		

BBIR_3_	Black *breu*	*Protium heptaphyllum* (Aubl.) Marchand	B	*p*-Cymene
WBB_1_	White *breu*	*Protium decandrum *(Aubl.) Marchand		

BBTF_1_	Black *breu* or *sucuruba*	*Protium* cf. *opacum *Swart	C	*p*-Cymene and high concentration of sesquiterpenes
BBTF_2_	Black *breu* or *sucuruba*	*Protium altsonii* Sandwith		

WBB_2_	White *breu*	*Protium occultum *D.C. Daly	D	Limonene and *α*-terpineol

WBIG	White *breu*	*Protium strumosum* Daly	E	*α*-Pinene

**Table 2:** Relative (%) chemical composition of the collected air after 1 and 15 minutes of nebulization of each formulation.

Formulations according to major compounds				A		B		C		D		E	
S.N.	Substance	RI_lit_^*∗*^	RI^*∗∗*^	Percentage (%)									
				1 min	15 min	1 min	15 min	1 min	15 min	1 min	15 min	1 min	15 min
(1)	*α*-Thujene	924	927	1.2	0.8	1.2	0.6	1.1	0.5	—	—	1.2	0.8
(2)	*α*-Pinene	932	934	4.3	1.9	19.2	8.5	3.7	1.7	5.2	—	**68.8**	**49.2**
(3)	Camphene	946	949	0.2	—	0.3	—	0.6	—	—	—	1.1	0.7
(4)	Verbenene	961	969	0.9	0.2	—	—	—	—	—	—	—	—
(5)	Sabinene	969	973	—	—	0.3	—	—	—	—	—	—	—
(6)	*trans*-*p*-Menthane	973	975	—	—	—	—	0.3	—	0.8	—	—	—
(7)	*β*-Pinene	974	980	—	—	3.5	1.0	—	—	—	—	9.5	7.7
(8)	2-Menthene^*∗*^	—	980	0.8	0.4	—	—	—	1.7	—	—	—	—
(9)	3-*p*-Menthene	984	984	—	—	—	—	1.1	0.7	—	—	—	—
(10)	Myrcene	988	990	0.8	0.5	—	0.6	—	—	—	—	—	—
(11)	Bornane^*∗*^	—	1001	—	—	2.1	1.9	—	—	—	—	—	—
(12)	*α*-Phellandrene	1002	1005	13.4	11.7	19.4	18.8	17.8	13.9	—	—	—	—
(13)	Mix (*δ*-3-carene and iso-sylvestrene)	1011	1011	**59.0**	**56.4**	16.7	16.7	2.7	2.6	2.7	2.5	—	—
(14)	*α*-Terpinene	1014	1017	0.9	0.5	3.6	3.7	7.2	5.8	—	—	0.5	0.6
(15)	1-*p*-Menthene	1021	1022	0.6	0.1	0.4	—	0.4	—	0.9	—	—	—
(16)	*p*-Cymene	1022	1026	8.2	7.1	**20.6**	**22.5**	**27.9**	**23.4**	18.8	6.6	5.3	6.2
(17)	Mix (limonene and *β*-phellandrene)	1024	1028	4.3	3.8	6.9	8.2	11.3	10.6	**33.4**	6.4	5.5	6.8
(18)	1,8-Cineole	1026	1031	1.6	1.1	—	—	—	—	—	—	—	—
(19)	*γ*-Terpinene	1054	1059	—	—	0.2	—	0.2	—	—	—	1.4	2.1
(20)	*m*-Cymenene	1082	1085	—	—	0.1	—	—	—	—	—	—	—
(21)	Terpinolene	1086	1088	0.3	0.2	—	0.7	0.2	0.8	1.1	—	0.3	0.4
(22)	*p*-Cymenene	1089	1092	—	—	0.2	—	—	—	—	—	—	—
(23)	Linalool	1098	1102	0.3	0.1	—	—	—	—	—	—	—	—
(24)	*cis*-*p*-Menth-2en-1-ol	1118	1123	—	—	0.1	—	—	—	—	—	—	—
(25)	Camphor	1141	1146	0.4	1.6	0.3	2.1	—	—	—	—	1.3	4.3
(26)	*trans*-Dihydro-*α*-terpineol	1143	1147	—	—	0.3	2.1	5.9	7.2	6.2	12.6	—	—
(27)	*cis*-Dihydro-*α*-terpineol	1164	1162	—	—	—	—	0.2	—	—	—	—	—
(28)	*p*-Mentha-1,5-dien-8-ol	1166	1167	0.4	1.8	—	—	—	—	—	—	—	—
(29)	Terpinen-4-ol	1174	1178	—	—	—	—	0.3	—	—	—	0.4	2.1
(30)	*p*-Cymen-8-ol	1179	1182	0.3	1.8	—	—	—	—	—	—	—	—
(31)	*α*-Terpineol	1186	1191	0.7	6.9	0.3	6.1	0.6	1.2	15.4	**68.9**	0.9	10.1
(32)	*γ*-Terpineol	1199	1199	—	—	—	—	—	—	1.0	—	—	—
(33)	*α*-Cubebene	1345	1351	—	—	—	—	1.0	3.3	—	—	—	—
(34)	Cyclosativene	1369	1370	—	—	—	—	—	1.1	—	—	—	—
(35)	*α*-Copaene	1374	1377	—	—	—	—	0.2	0.7	—	—	—	—
(36)	Cyperene	1398	1398	—	—	—	—	0.3	0.8	—	—	—	—
(37)	*α*-Cedrene	1410	1414	—	—	—	—	0.2	—	—	—	—	—
(38)	*α*-*cis*-Bergamotene	1411	1416	—	—	—	—	—	—	—	—	0.2	2.2
(39)	*β*-Caryophyllene	1417	1423	—	—	—	—	0.5	1.6	—	—	—	—
(40)	*β*-Cedrene	1419	1420	—	—	—	—	0.5	1.6	—	—	—	—
(41)	*trans*-*α*-Bergamotene	1432	1437	—	—	—	—	0.4	1.4	—	—	—	—
(42)	*α*-Guaiene	1437	1444	—	—	—	—	0.2	—	—	—	—	—
(43)	Aromadendrene	1439	1448	—	—	—	—	0.1	0.4	—	—	—	—
(44)	*β*-Barbatene	1440	1445	—	—	—	—	0.1	0.4	—	—	—	—
(45)	*α*-Neo-clovene	1452	1455	—	—	—	—	0.4	1.2	—	—	—	—
(46)	Khusimene	1453	1454	—	—	—	—	0.4	1.2	—	—	—	—
(47)	*α*-Neocallitropsene	1474	1481	—	—	—	—	0.5	1.8	—	—	—	—
(48)	*γ*-Gurjunene	1475	1480	—	—	—	—	0.5	1.8	—	—	—	—
(49)	*γ*-Muurolene	1478	1478	—	—	0.1	1.9	0.2	—	—	—	—	—
(50)	Germacrene D	1480	1483	—	—	—	—	0.1	—	—	—	—	—
(51)	*cis*-*β*-Guaiene	1492	1496	—	—	—	—	0.1	—	—	—	—	—
(52)	*trans*-*β*-Guaiene	1502	1513	—	—	—	—	0.1	0.4	—	—	—	—
(53)	Cuparene	1504	1509	—	—	—	—	0.1	0.4	—	—	—	—
(54)	*γ*-Cadinene	1513	1515	—	—	—	—	1.7	6.2	—	—	—	—
(55)	*δ*-Cadinene	1522	1525	—	—	—	—	0.3	1.2	—	—	—	—
(56)	(*E*)-*γ*-Bisabolene	1529	1533	—	—	—	—	—	—	—	—	0.1	2.5
(57)	*trans*-Cadina-1,4-diene	1533	1541	—	—	—	—	0.3	1.2	—	—	—	—
(58)	1,10-di-*epi*-Cubenol	1618	1620	—	—	—	—	0.3	—	—	—	—	—
Monoterpene hydrocarbons		—	—	96.5	84.7	94.7	83.2	74.5	61.7	62.9	15.5	93.6	74.5
Oxygenated monoterpenes		—	—	2.1	12.1	1.0	10.3	7.0	8.4	22.6	81.5	2.6	16.5
Sesquiterpene hydrocarbons		—	—	—	—	0.1	1.9	8.2	26.7	—	—	0.3	4.7
Oxygenated sesquiterpenes		—	—	—	—	—	—	0.3	—	—	—	—	—

Components listed in order of elution of the HP-5 column. ^*∗*^Tentative identification. Bold characters represent major compounds. S.N. means substance number. Monoterpene hydrocarbons: S.N. (1)–(22); oxygenated monoterpenes: S.N. (23)–(32); sesquiterpene hydrocarbons: S.N. (33)–(57); oxygenated sesquiterpenes: S.N. (58). RI_lit_^*∗*^, retention indices obtained in the literature; RI^*∗∗*^, linear retention indices calculated from a homologous series of n-alkanes C_7_–C_26_. Percentage was obtained by normalizing the FID peaks area.

**Table 3:** Effects of formulations on hot plate test in mice.

Treatment	Latency period (s)	
	5 min	30 min
Compressed air	7.3 ± 2.2	6.5 ± 1.2
Vehicle	4.8 ± 0.7	4.9 ± 0.9
Formulation A	5.0 ± 1.6	5.7 ± 2.0
Formulation B	3.9 ± 0.5	3.9 ± 0.6
Formulation C	4.7 ± 1.1	4.8 ± 1.3
Formulation D	7.6 ± 3.0	8.6 ± 4.4
Formulation E	4.8 ± 0.1	6.8 ± 1.5

Each value is presented as the mean ± SEM (*n* = 5).

**Table 4:** Effects of formulations on formalin-induced paw licking in mice.

Treatment	Licking of the hind paw (s)			
	Early phase (0–5 min)	% Inhibition	Late phase (15–30 min)	% Inhibition
Compressed air	72.0 ± 10.0	—	172.80 ± 6.5	—
Vehicle	65.40 ± 8.4	—	156.60 ± 10.5	—
Formulation A	59.40 ± 5.2	9.17	134.20 ± 21.2	14.3
Formulation B	41.30 ± 4.3	36.9^*∗*^	159.30 ± 37.0	0.0
Formulation C	48.80 ± 4.3	25.4	146.70 ± 36.2	6.3
Formulation D	57.40 ± 9.3	12.2	97.20 ± 6.6	37.9^*∗*^
Formulation E	34.80 ± 3.8	46.8^*∗*^	62.40 ± 8.3	60.2^*∗*^

Each value is presented as the mean ± SEM (*n* = 5); *∗* indicates *p* < 0.05 compared with vehicle group (Bonferroni's test).

**Table 5:** Effects of formulations on rota-rod test in mice.

Treatment	Number of falls	
	0 min	30 min
Compressed air	0 ± 0	0 ± 0
Vehicle	0.4 ± 0.5	0 ± 0
Formulation A	0.8 ± 0.8	0.2 ± 0.4
Formulation B	0.4 ± 0.9	0.3 ± 0.4
Formulation C	0.2 ± 0.4	0 ± 0
Formulation D	0.6 ± 0.9	0 ± 0
Formulation E	0.4 ± 0.5	0.2 ± 0.4

Each value is presented as the mean ± SEM (*n* = 5).
